# *Baylisascaris procyonis* Roundworm Infection in Child with Autism Spectrum Disorder, Washington, USA, 2022

**DOI:** 10.3201/eid2906.230290

**Published:** 2023-06

**Authors:** Beth A. Lipton, Hanna N. Oltean, Roger B. Capron, Arran Hamlet, Susan P. Montgomery, Rebecca J. Chancey, Victoria J.L. Konold, Katherine E. Steffl

**Affiliations:** Washington State Department of Health, Shoreline, Washington, USA (B.A. Lipton, H.N. Oltean, A. Hamlet);; Skagit County Health Department, Mount Vernon, Washington, USA (R.B. Capron);; Centers for Disease Control and Prevention, Atlanta, Georgia, USA (A. Hamlet, S.P. Montgomery, R.J. Chancey);; Seattle Children’s Hospital, Seattle, Washington, USA (V.J.L. Konold, K.E. Steffl)

**Keywords:** *Baylisascaris procyonis*, Ascaridida infections, Ascaridoidea, roundworm, case report, raccoons, feces, larva migrans, eosinophilia, meningitis/encephalitis, albendazole, child, autism spectrum disorder, intellectual disability, pica, parasites, zoonoses, Washington, United States

## Abstract

We describe a case of *Baylisascaris procyonis* roundworm infection in a child in Washington, USA, with autism spectrum disorder. Environmental assessment confirmed nearby raccoon habitation and *B*. *procyonis* eggs. *B*. *procyonis* infections should be considered a potential cause of human eosinophilic meningitis, particularly among young children and persons with developmental delays.

*Baylisascaris* spp. are ascarid worms that parasitize the small intestines of multiple species. The primary definitive host for *B. procyonis *roundworms is the raccoon (*Procyon lotor*), although other carnivores, including dogs, can serve as definitive hosts (*1*). Infected raccoons shed >1 million *B. procyonis* eggs daily; excreted eggs take 2–4 weeks to embryonate and become infective. In the western United States, the estimated prevalence of *B. procyonis* infection in raccoons is 68%–82% (*2*). Raccoons often defecate in communal locations (raccoon latrines) that are close to areas of human activity or living spaces, such as yards, decks, roofs, or attics. Humans become intermediate hosts after ingesting infective eggs that hatch into larvae, penetrate the gut wall, and migrate through tissues, potentially resulting in visceral, ocular, or neural larva migrans syndromes; neural larva migrans often manifests as acute eosinophilic meningitis. Tissue damage and clinical outcomes of baylisascariasis might be severe; permanent neurologic sequalae and death might occur because of the large size of *B. procyonis* larvae and invasive tissue migration (*3*). Severity of disease is related to host size, number of eggs ingested, larval migration pathway, and extent of host inflammatory responses (*4*,*5*). As with other parasite larva migrans, *B*.* procyonis* larvae are found mostly in muscle tissue; <5% of migrating larvae reach the brain (*5*). Asymptomatic and subclinical infections are known to occur. Fewer ingested eggs might result in positive serologic tests in the absence of neurologic disease (*5*,*6*).

We describe a case of *B. procyonis* infection in a child in Washington, USA, with autism spectrum disorder and history of pica who had eosinophilic meningitis. We conducted an environmental assessment of the patient’s residence to assess raccoon activity and potential exposure sources. 

## The Study

In July 2022, a 7-year-old boy with a history of autism spectrum disorder and global developmental delay accompanied by lack of verbal ability began having episodes of mild motor impairment, lethargy, decreased responsiveness, and difficulty understanding and executing commands that lasted 45 minutes to 2 hours (first episode = day 0). He was seen at an emergency department on day 11, then hospitalized by a second emergency department the next day after a prolonged period of stumbling and lethargy. The patient had not traveled outside of Washington in the past 2 years and had no household pets. In recent weeks, the patient had played in a sandbox where nonhuman feces were observed and visited a location with farm animals.

**Figure 1:**
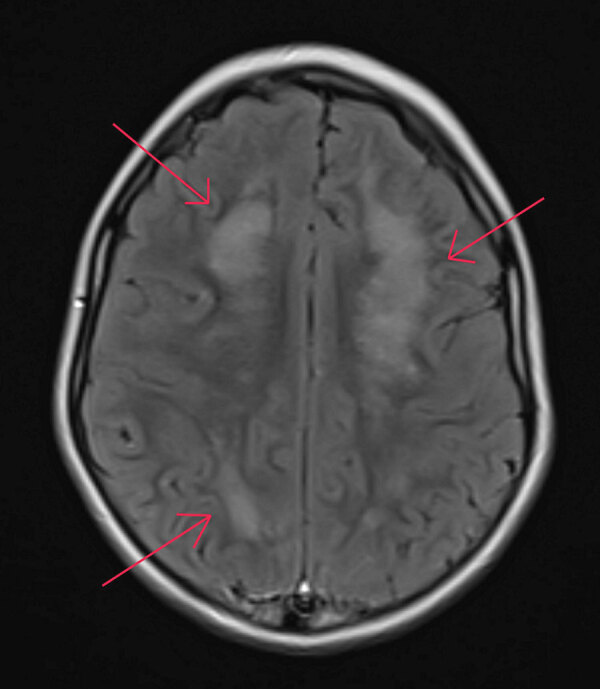
Magnetic resonance imaging of brain of child with autism spectrum disorder infected with *Baylisascaris procyonis* roundworms, Washington, USA, 2022. Axial section of the brain shows patchy white matter disease with limited gray matter involvement. Red arrows indicate diseased regions.

While hospitalized, the patient underwent several tests. A complete blood count showed peripheral eosinophilia (1,693 eosinophils/µL), and results of an upper respiratory virus and bacteria PCR panel were negative. An electroencephalogram indicated focal cerebral pathology, and brain magnetic resonance imaging showed patchy white matter disease with limited gray matter involvement (Figure 1). Cerebrospinal fluid (CSF) was collected and had negative PCR results for cytomegalovirus, enterovirus, herpes simplex virus 1, herpes simplex virus 2, herpes simplex virus 6, human parechovirus, and varicella zoster virus. CSF was Gram stain and culture negative but had a nucleated cell count of 6 cells/μL (reference range 0–5 cells/μL) with 50% eosinophils. Acute disseminated encephalomyelitis was suspected, and corticosteroid treatment was initiated. Consultation with the Centers for Disease Control and Prevention (CDC) was pursued, given the eosinophilic meningitis and possible parasitic cause of illness, after which a 10-day course of albendazole (25 mg/kg body weight/d) was initiated on day 17. 

The patient’s serum and CSF specimens were sent to CDC for *B. procyonis* testing. The patient’s serum was positive for *B. procyonis*–specific antibodies; CSF was antibody negative. A *Toxocara* spp. IgG test was positive, although false-positive results might occur in patients with other parasitic infections, including *Baylisascaris* spp*.* Conversely, *B. procyonis* test cross-reactivity with toxocariasis was not expected (*7*). On day 23, the patient was discharged from the hospital. The family reported ongoing but improving symptoms 1 month after discharge.

**Figure 2:**
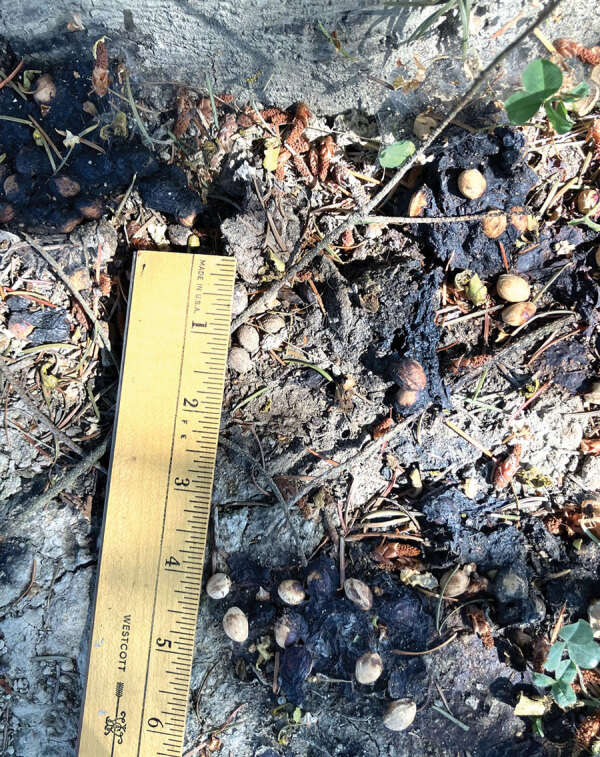
Raccoon latrine found at residence of child with autism spectrum disorder infected with *Baylisascaris procyonis* roundworms, Washington, USA, 2022.

In mid-September 2022, we performed an environmental investigation at the patient’s residence. The family had never seen raccoons in the yard but noted possible animal feces at the base of a fir tree. After additional questioning, the family recalled that the patient had put material from the ground around this tree in his mouth in July; we observed a raccoon latrine at this location (Figure 2). We collected 4 fecal samples for fecal flotation analysis from the following locations: raccoon latrine (location 1, 2 samples), a grassy area away from the latrine (location 2, 1 sample), and the sandbox (location 3, 1 sample). Two samples were positive for *B. procyonis* eggs, and 2 samples were positive for non*–B. procyonis* roundworm eggs (Table).

**Table:** Fecal flotation results from environmental sampling in September 2022 at residence of child with autism spectrum disorder infected with *Baylisascaris procyonis* roundworms, Washington, USA*

Fecal sample characteristics	Organism	Result	No. eggs/g†
Location 1, pooled sample 1, raccoon latrine at base of fir tree	*B. procyonis*	Positive	14
	Non-*B. procyonis* roundworms	Negative	NA
Location 1, pooled sample 2, raccoon latrine at base of fir tree	*B. procyonis*	Negative	NA
	Non-*B. procyonis* roundworms	Negative	NA
Location 2, unidentified feces in grassy area away from raccoon latrine	*B. procyonis*	Positive	25
	Non-*B. procyonis* roundworms	Positive	1
Location 3, pooled sample, suspected cat feces in sandbox	*B. procyonis*	Negative	NA
	Non-*B. procyonis* roundworms	Positive	1

*NA, not applicable. †No. eggs per gram of collected feces detected by fecal flotation.

We noted various raccoon attractants around the property, including bushes and trees, a brush pile, and a large open shed. We provided oral feedback and written materials regarding safe latrine clean-up, regular observation and prompt clean-up of feces, and methods to discourage raccoons from inhabiting nearby areas. Raccoons are not a reservoir species for rabies in Washington; therefore, we did not provide education on rabies prevention.

Reported cases of human *B. procyonis* infection are rare, despite the proximity and prevalence of infected raccoons across much of the United States (*8*). We found 37 published cases of *B. procyonis* infection in North America (33 in the United States, 4 in Canada) (*2*,*4*,*8*–*13*). Of published case reports, including this study, the median age of infected persons was 1.6 years (range 9 months–73 years); 32 (82%) persons were male, and 7 (18%) infections resulted in death. Geophagia or pica was mentioned in 21 (55%) cases, and 14 (37%) cases described developmental disabilities in the patients. Case-patients with geophagia or pica were associated with an increased risk for death, possibly because of ingestion of higher numbers of *B. procyonis* eggs. All but 2 cases described neurologic symptoms. Undetected subclinical infections and lack of commercially available testing likely leads to overestimation of the overall death rate (*5*–*7*). Baylisascariasis is not nationally notifiable in the United States but was recently added to the notifiable conditions list in Washington (*14*).

## Conclusions

Young children and persons with developmental delays are at high risk for *B. procyonis* infection because of hand–mouth behaviors, as are persons exposed to raccoons or environments where raccoons are frequently found (*3*). Healthcare providers should consider *B. procyonis* roundworm infections a possible cause of eosinophilic meningitis even without known patient exposure to raccoon feces. The ubiquitous nature of raccoons and the 1–4-week incubation period for infection might cause difficulty in characterizing potential exposure locations. Treatment with albendazole and corticosteroids during the early stage of infection might reduce serious tissue damage, although no treatments are totally effective (*3*,*9*). Known or suspected oral exposure to raccoon feces indicates immediate prophylactic treatment with albendazole should be considered, which might prevent disease (*10*).

Raccoon latrines have diverse locations, sizes, and appearances. Raccoons might share multiple latrines over a short period of time, increasing the accumulation of *B*. *procyonis* eggs (*15*). Feces are often dark and tubular; latrines might remain active for >1 year (*3*,*15*). Eggs can remain viable in the environment for years and are difficult to kill or remove. CDC provides resources on how to identify and safely clean up a latrine (*3*).

In summary, discouraging raccoons from residential areas and increased recognition of raccoon latrines can help prevent *B. procyonis* infections. Public and healthcare provider awareness of *B. procyonis* infection risks, especially for persons at higher risk such as young children and those with developmental delays, is critical for prevention and early treatment and improving disease outcome. 
